# Occurrence of Horizontal Gene Transfer of P_IB_-type ATPase Genes among Bacteria Isolated from the Uranium Rich Deposit of Domiasiat in North East India

**DOI:** 10.1371/journal.pone.0048199

**Published:** 2012-10-25

**Authors:** Macmillan Nongkhlaw, Rakshak Kumar, Celin Acharya, Santa Ram Joshi

**Affiliations:** 1 Microbiology Laboratory, Department of Biotechnology and Bioinformatics, North-Eastern Hill University, Umshing, Shillong, Meghalaya, India; 2 Molecular Biology Division, Bhabha Atomic Research Centre, Trombay, Mumbai, Maharashtra, India; Belgian Nuclear Research Centre SCK/CEN, Belgium

## Abstract

Uranium (U) tolerant aerobic heterotrophs were isolated from the subsurface soils of one of the pre-mined U-rich deposits at Domiasiat located in the north-eastern part of India. On screening of genomic DNA from 62 isolates exhibiting superior U and heavy metal tolerance, 32 isolates were found to be positive for P_IB_-type ATPase genes. Phylogenetic incongruence and anomalous DNA base compositions revealed the acquisition of P_IB_-type ATPase genes by six isolates through horizontal gene transfer (HGT). Three of these instances of HGT appeared to have occurred at inter-phylum level and the other three instances indicated to have taken place at intra-phylum level. This study provides an insight into one of the possible survival strategies that bacteria might employ to adapt to environments rich in uranium and heavy metals.

## Introduction

Domiasiat (25° 30′ N 91° 30′ E) represents one of the pre-mined uranium rich areas located in Meghalaya in the north-eastern part of India. Exploration by the Atomic Mineral Division (AMD) of India in 1986 led to the discovery of sandstone type uranium deposits occurring at shallow depth. As is the case for other U-ore bearing sites, Domiasiat co-hosts additional heavy metals including zinc, copper, cadmium and lead [1]. Metals may influence the growth and occurrence of organisms, either as limiting factors or through toxicity. Bacteria existing in metal rich/contaminated soils are known to have evolved strategies to negate the toxic effects of metals. One such mechanism relates to the maintenance of metal homeostasis within the cell, usually accomplished by the active efflux of metal ions [2]. P_IB_-type ATPases are transmembrane proteins that are known to govern the transport of heavy metals such as Cu, Zn, Pb and Cd in bacteria [3], [4]. These transport mechanisms use energy from ATP hydrolysis to pump out metal ions from the cytoplasm towards the periplasm [3], [4]. Although P_IB_-type ATPases are mostly chromosomally encoded, some studies have reported the presence of PIB-type ATPase genes on mobile genetic elements (i.e. plasmids and transposons) in both gram-positive bacteria [5]–[8] and gram-negative bacteria [9], [10]. Based on substrate specificity, members of the P-type ATPase proteins are classified into distinguishable families [11]. Furthermore, the P_IB_-type sub-family tends to separate into two distinct groups based on the cation they transport, i.e. (a) those specific to monovalent cations like Cu^+^ or Ag^+^ and (b) those transporting divalent cations like Zn^2+^, Cd^2+^, Co^2+^ and Pb^2+^ [3], [11]. So far, no signature motif has been identified that suggests the substrate specificity in these proteins. Further biochemical characterization is therefore required to delineate the metal specificity in these proteins.

Horizontal gene transfer (HGT) is considered to be a key factor in providing adaptive features to microbes for their survival and proliferation by defining the structure and function of microbial communities in their natural habitats [12], [13]. It has been shown to be prevalent in the prokaryotic genome with low frequencies of recombination and is considered to be responsible for generating diversity and adaptability among microorganisms [14]. While the role of HGT has been well documented for the transfer of antibiotic resistance and pathogenic genes in bacteria [15], its contribution to the transfer of metal transporting genes is being recently explored. Genome wide studies by Coombs and Barkay [16] showed that HGT of metal-transporting P_IB_-type ATPase encoding genes can occur albeit at a low frequency. Molecular evidence for HGT was observed among lead-resistant bacteria isolated from deep subsurface environments contaminated with radionuclides and heavy metals [17], [18].

In the present study, we isolated uranium tolerant aerobic heterotrophs from subsurface soils of one of the uranium rich deposits located at Domiasiat in north eastern part of India. We examined the presence of P_IB_-type ATPase genes in these uranium tolerant bacterial isolates. Six isolates belonging to *Bacteroidetes*, *Firmicutes*, and *Proteobacteria* phyla, among the population of P_IB_-type ATPase positive isolates, revealed the acquisition of *zntA/cadA/pbrA*-like genes through HGT.

## Materials and Methods

### Experimental site and soil samples

Soil samples were collected from the proposed mining site of one of the U ore deposits at Domiasiat (25° 30′ N 91° 30′ E) in North-East India. This site formed a part of a Cretaceous Mahadek basin covering roughly an area of 13,000 km^2^ containing 9.22 million metric tonnes of ore reserves with an average ore grade of circa 0.1% U_3_O_8_ [19]. The soil samples contained variable concentrations of U ranging from 40 μM to 5 mM (unpublished data). The pH of the soil was acidic within the range of 4.3 to 6.3 [20].

### Isolation of uranium tolerant bacteria

Duplicate (subsurface) soil samples (10 g) were inoculated in 100 mL of low phosphate medium (LPM) [21] (pH 7.5) in Erlenmeyer flasks amended with 1 mM U(VI) as [UO_2_ (NO_3_)_2_. 6H_2_O] and incubated at 30°C at 150 rpm for 48 h. Serial ten-fold dilutions of these enrichment cultures were inoculated onto the LPM agar (pH 7.0) plates supplemented with 1 mM of U(VI) to isolate U-tolerant populations. Plates were incubated at 30°C for 72 h [22].

### Determination of the minimum inhibitory concentration (MIC) for U and other heavy metals

Analytical grade salts of uranyl nitrate [UO_2_(NO_3_)_2._6H_2_O] (Merck), copper sulphate [CuSO_4_.5H_2_O], cadmium nitrate [Cd(NO_3_)_2_.5H_2_O] and lead nitrate [Pb(NO_3_)_2_] (Himedia, India), were used to prepare stock solutions of the respective metals. These solutions were filter sterilized through a 0.22 µm nitrocellulose membrane filter (Millipore, India). The representative isolates including the type strains *Serratia marcescens* ATCC13880, *Bacillus lichenoformis* MTCC429, *Bacillus cereus* MTCC430, *Arthrobacter ureafaciens* MTCC3454, *Sphingobacterium multivorum* MTCC498, and *Escherichia coli* MTCC118 were grown to mid-exponential phase in low phosphate medium (LPM) containing (g L^−1^): Tris, 14.5; NaC1, 4.68; KCI, 1.5; NH_4_Cl, 1.0; glycerol, 5; Na_2_S0_4_, 0.043; CaCl_2_, 0.03 (pH adjusted to 7.5 with concentrated HCl) [21]. The cells were then washed twice with 0.9% NaCl. 10 µL of the cell suspension of each isolate was spotted onto LPM agar medium plates (150 mm diameter) [23] and incubated at 30°C for 48 h. The MIC was defined as the minimum concentration of metal that inhibited visible growth of the inoculated culture after 2 days of incubation at 30°C [24], [25].

### Detection of P_IB_-type ATPase genes

The bacterial isolates were screened for the presence of metal transporting P_IB-_type ATPase (e.g., *zntA/cadA/pbrA*-like) genes. Genomic DNA was isolated from the respective isolates using genomic DNA extraction kit (HiMedia, India) and amplified with PCR primers specific for P_IB_-type ATPase encoding genes [17], [18]. PCR mixtures (25 μL) contained approximately 30 ng of template DNA, 2 μM forward primer (81JC or 133JC), 2 μM reverse primer (84JC), Taq DNA Polymerase buffer with 15 mM MgCl_2_, deoxynucleoside triphosphates (250 μM each of dATP, dCTP, dGTP and dTTP) and 1.0 U of Taq DNA polymerase. DNA amplification was carried out in a GeneAmp® PCR system 9700 (Applied Biosystems, USA) with an initial denaturation step of 94°C for 5 min, followed by 30 cycles consisting of denaturation at 94°C for 1 min, annealing at 49°C for 1 min, and extension at 72°C for 1.5 min followed by a final extension step of 72°C for 5 min. This PCR procedure yielded amplicons of the expected size (∼750 bp).

### Phylogenetic analyses of *zntA*/*cadA*/*pbrA*-like gene products and 16S rRNA genes

Amplicons of P_IB_-type ATPase genes (*zntA/cadA pbrA*-like) of the representative 16 isolates from different phyla were purified using the QIAquick Gel Extraction Spin Kit (QIAGEN, Germany) and sequenced using the Big Dye Terminator cycle sequencing kit v.3.1 (Applied Biosystems, USA) deploying the standard protocol and an automated Genetic Analyzer ABI 3130XL (Applied Biosystems, USA). The Basic Local Alignment Search Tool (BLAST, sub-program BLASTX) [26] was used to determine the phylogenetic neighbours of P_IB_-type ATPase genes against the GenBank database (National Center for Biotechnology Information, Bethesda, USA). Molecular Evolutionary Genetics Analysis software (MEGA v4) was used for phylogenetic analyses [27]. The sequences of identified phylogenetic neighbours were aligned with the sequences using ClustalW of MEGA4. The *Escherichia coli kdpB* gene was used as outlier. For phylogenetic tree construction we used the neighbor joining method with 1000 bootstrap replications for nodal support. Phylogenetic analyses based on the maximum likelihood and maximum parsimony of P_IB_-type ATPase amino acid sequences were in agreement with the data generated by the above described neighbour joining method. On average, 600 nucleotides were included in the phylogenetic analyses of the P_IB_-type ATPase sequences. The 16S rRNA gene sequences of the representative bacteria were amplified and sequenced as previously described [28]. Phylogenetic neighbours were obtained against the database of type strains with validly published prokaryotic names (available online http://eztaxon-e.ezbiocloud.net/) [29]. Phylogenetic analyses by MEGA4 were performed using an average of 1200 nucleotides of 16S rRNA encoding DNA sequence [27]. The 16S rRNA gene sequence of *Deinococcus radiodurans* M21413 was taken as an outlier. The phylogenetic tree was constructed using the scale bars of 0.05 change per nucleotide position for the 16S rRNA gene and 0.1 change per amino acid position for PIB-type ATPase.

The G+C content of each *zntA*/*cadA*/*pbrA*-like amplicon was calculated by using Oligo Calculator available at http://mcb.berkeley.edu/labs and were compared to the G+C contents of all other organisms belonging to the same genus.

### Nucleotide sequence accession numbers

**Figure 1:**
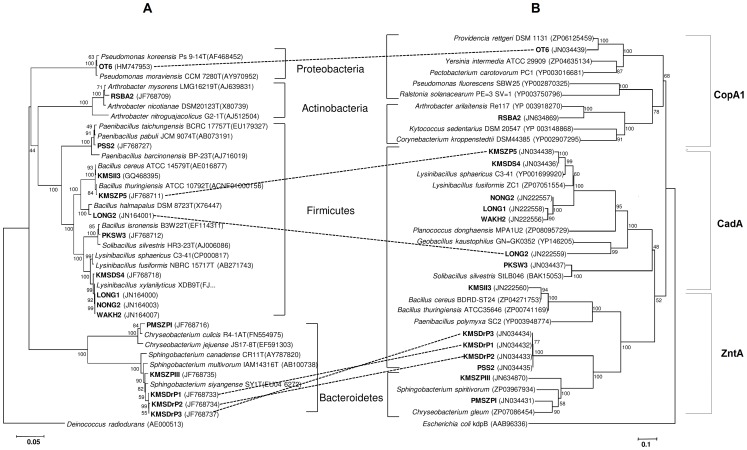
Molecular evidence for horizontal gene transfer among Domiasiat isolates. The genes encoding (A) 16S rRNA and (B) *zntA/cadA/pbrA*-like transporters.of uranium and heavy metal tolerant isolates obtained from subsurface soils of U-rich deposits of the Domiasiat site were subjected to neighbor-joining analysis. Respective accession numbers of gene nucleotide sequences are indicated in brackets. P_IB_-type ATPase positive isolates predicted to have undergone HGT are connected by dotted lines. The scale bars indicate 0.05 change per nucleotide position for the 16S rRNA gene and 0.1 change per amino acid position for P_IB_-type ATPase phylogeny.

Nucleotide sequences of 16S rRNA and P_IB_-type ATPase encoding genes have been deposited in NCBI with accession numbers as shown in Figure 1A and 1B, respectively.

## Results

### Tolerance to uranium and heavy metals among natural bacterial isolates and analysis of P_IB_-type ATPase genes

One hundred and thirty uranium tolerant isolates were obtained from LPM plates enriched with uranium. Out of those, 62 isolates were selected for further studies based on amplified ribosomal DNA restriction analysis (ARDRA) grouping (data not shown). The natural isolates from Domiasiat were able to tolerate significant concentrations of U (2mM) and other heavy metals (data not shown). These metal-tolerant isolates were screened for the presence of PIB-type ATPase (*zntA/cadA/pbrA*-like) encoding genes using primers reported in earlier investigation [18]. Out of the 62 isolates analysed for the presence of P_IB_-type ATPase encoding genes, 32 isolates tested positive and yielded gene products of the expected size of ∼750 bp. Based on the superior metal tolerance potential, 16 out of 32 PCR positive isolates (representing each genus), were selected for further sequence analysis. Fourteen of these isolates showed PCR amplifiable *zntA*/*cadA*/*pbrA* loci whereas the other two isolates displayed *copA*-like loci. The amino acid sequences deduced from the PIB-type ATPase gene sequences of seven isolates, i.e. WAKH2, NONG2, LONG1, KMSDS4, RSBA2, PMSZPI and KMSII3 showed ≥80% similarity with the PIB-type ATPase signature sequences of the expected genus in the GenBank database (Table 1). The deduced PIB-type amino acid sequences of the other nine isolates either showed close similarity with another genus or displayed poor similarity (<70%) with the corresponding genus. The first closest match revealed by phylogenetic analysis of PIB-type ATPases for the isolates PKSW3 and RSBA2 indicated cation transporting ATPases while the second match corresponded to Cd and Cu transporting genes, respectively (Table 1). Neighbour joining analyses of gene sequences of natural isolates were performed with the nearest homologous *zntA/cadA/copA*-like loci from complete genomes available at NCBI GenBank. The combined phylogenetic clustering with the PCR amplified gene sequences for PIB-type ATPases obtained from Domiasiat isolates corresponded to ZntA-like, CopA-like, and CadA-like major clusters (Figure 1B). The phylogenetic tree created using the CopA-like loci of OT6 and RSBA2 along with the corresponding sequences from complete genomes from NCBI (as identified by sequence similarity using BLASTX) resulted in CopA1-like cluster (Figure 1B, Figure S1). This is in agreement with previous reports indicating the coherence between the substrate specificity and phylogeny of P-type ATPases [11]. The MICs of U and other heavy metals like Cd, Cu and Pb for the 16 representative bacteria are shown in Table 1. All the isolates exhibited a MIC of 4 mM for U against 1–2 mM shown by type strains. Half (50%) of the bacterial isolates showed a MIC of 8 mM for Cu against 2–4 mM demonstrated by the type strains. For Cd, 56% of the isolates displayed a MIC of 2 mM while 44% showed a MIC of 1 mM as compared to a MIC of 60–500 μM for Cd by the type strains. While one half of the population exhibited a MIC of 2 mM for Pb, the other half showed a MIC of 1 mM against 500 μM shown by the tested type strains (Table 1). These results confirmed the superior metal tolerance phenotypes of the natural isolates of Domiasiat harbouring PIB-type ATPases.

**Table 1:** Comparative matches for the closest phylogenetic neighbours obtained for the isolates based on profile of 16S rRNA gene and P_IB_-type ATPases gene.

Isolates	Closest match of 16S rRNA gene with similarity percentage	Closest match of P_IB_-type ATPases gene with similarity percentage	MIC (millimolar)			
			U	Cu	Cd	Pb
KMSII3	*Bacillus cereus*, **100%**	Zn-transporting ATPase, *Bacillus thuringiensis*, **99%**	4.0	4.0	1.0	1.0
LONG2	*Bacillus halmapalus*, **99.07%**	Cd-transporting ATPase, *Geobacillus kaustophilus*, **64%**	4.0	8.0	1.0	1.0
KMSZP5	*Bacillus thuringiensis*, **99.86%**	Cd-transporting ATPase, *Lysinibacillus sphaericus*, **94%**	4.0	4.0	1.0	1.0
PKSW3	*Bacillus isronensis*, **99.58%**	Cation transport ATPase, *Solibacillus silvestris*, **94%** Cd-transporting ATPase, *Lysinibacillus sphaericus*,**67%**	4.0	8.0	2.0	2.0
KMSDS4	*Lysinibacillus xylanilyticus*, **99.62%**	Cd-transporting ATPase, *Lysinibacillus sphaericus*, **95%**	4.0	4.0	2.0	1.0
LONG1	*Lysinibacillus xylanilyticus*, **100%**	Cd-transporting ATPase, *Lysinibacillus fusiformis*, **82%**	4.0	8.0	2.0	2.0
NONG2	*Lysinibacillus xylanilyticus*, **100%**	Cd-transporting ATPase, *Lysinibacillus fusiformis*, **85%**	4.0	4.0	1.0	1.0
WAKH2	*Lysinibacillus xylanilyticus*, **100%**	Cd-transporting ATPase, *Lysinibacillus fusiformis*, **81%**	4.0	4.0	2.0	1.0
PSS2	*Paenibacillus pabuli*, **99.45%**	Zn-exporting ATPase, *Sphingobacterium spiritivorum*, **66%**	4.0	4.0	1.0	1.0
RSBA2	*Arthrobacter mysorens*, **99.02%**	Cation-transporting ATPase, *Arthrobacter* *arilaitensis*, **78%** Cu-translocating P-type ATPase, *Arthrobacter aurescens*, **55%**	4.0	4.0	1.0	1.0
OT6	*Pseudomonas koreensis*, **99.57%**	Cu-transporting P-type ATPase, *Providencia rettgeri*, **92%**	4.0	8.0	2.0	2.0
PMSZPI	*Chryseobacterium culicis*, **98.87%**	Zn-exporting ATPase, *Chryseobacterium gleum*, **92%**	4.0	4.0	1.0	2.0
KMSDrP1	*Sphingobacterium siyangense*, **99.02%**	Zn-exporting ATPase, *Sphingobacterium spiritivorum*, **66%**	4.0	8.0	2.0	2.0
KMSDrP2	*Sphingobacterium siyangense*, **99.01%**	Zn-exporting ATPase, *Sphingobacterium spiritivorum*, **66%**	4.0	8.0	2.0	2.0
KMSDrP3	*Sphingobacterium siyangense*, **98.96%**	Zn-exporting ATPase, *Sphingobacterium spiritivorum*, **66%**	4.0	8.0	2.0	2.0
KMSZPIII	*Sphingobacterium siyangense*, **99.03%**	Zn-exporting ATPase, *Chryseobacterium gleum*, **76%**	4.0	8.0	2.0	2.0

### Horizontal transfer of P_IB_-type ATPase genes

Phylogenetic incongruence between a gene of interest and a marker gene such as the 16S rRNA encoding gene has been used in earlier studies to predict HGT during gene evolution [30], [31]. BLASTX analysis was performed using *zntA/cadA/pbrA*-like sequences obtained from our isolates to find out the closest relative from the NCBI database. The similarity percentage of some of the *zntA/cadA/pbrA*-like sequences was found to be lower (<70%) as compared to the corresponding gene from the same genus reported in the NCBI database (Table 1). Six of our isolates appeared to have acquired *zntA/cadA/pbrA*-like genes by HGT (Figure 1, Table S1). The P_IB_-type ATPase gene sequences from *Sphingobacterium* sp. isolates, KMSDrP1, KMSDrP2, and KMSDrP3, exhibited circa 66% similarity with the *zntA* gene of *Sphingobacterium spiritivorum* ATCC33861 (as derived from its complete genome sequence; NCBI reference sequence, NZ_ACHA02000002.1(Table 1). However, these isolates showed 100% similarity with the *zntA* like sequence of *Paenibacillus pabuli* PSS2 isolated from Domiasiat site and clustered with the *zntA*-like gene cluster of *Firmicutes* and *Bacteroidetes* unlike the clusters obtained using 16S rRNA gene sequence analysis (Figure 1A, B). Initial analysis suggested the occurrence of HGT from *Bacteroidetes* (*Sphingobacterium* isolates) to the *Firmicutes* (*Paenibacillus* isolate). However, on inclusion of another important criterion for implicating HGT, *i.e*., moles percent (mol%) of G+C content (atypical sequence composition) [32], HGT appeared to have taken place from *Firmicutes* to *Bacteroidetes*. The G+C content of the *zntA*-like gene sequences from three *Sphingobacterium* isolates (47.3 mol%) was clearly higher than the DNA G+C content reported so far for *Sphingobacterium* species (i.e., 37.3 to 44.2 mol%) [33]. Furthermore, the G+C contents of *zntA* like gene sequences of the *Sphingobacterium* isolates were found to be almost similar to that of PSS2 identified as *Paenibacillus pabuli* by 16S rRNA gene phylogeny. The G+C content of the *Paenibacillus pabuli* genome is reported to be 45.3 mol% [34] which compares well to that of the *zntA* sequences of the isolates KMSDrP1(47.4% mol G+C), KMSDrP2(47.3% mol G+C), KMSDrP3 (47.3% mol G+C), and PSS2 (47.5% mol G+C), indicating HGT from *Firmicutes* to *Bacteroidetes*. In addition to a resemblance in mol% G+C contents, an incongruence in the phylogenetic tree was clearly observed when *zntA* like sequences of other *Bacteroidetes* isolates, i.e. PMSZPI and KMSZPIII, were excluded (Figure S2). However, on inclusion of representatives of all phyla, we found that the *Sphingobacterium* isolates (KMSDrP1, KMSDrP2, and KMSDrP3) and *Firmicutes* isolate (PSS2) clustered with both *Bacteroidetes* and *Firmicutes* with a strong bootstrap support of 100% (Figure 1B). As the mol% G+C content of PSS2, KMSDrP1, KMSDrP2, and KMSDrP3 corresponded well to that of *Firmicutes*, we have assigned these as *Firmicutes* rather than *Bacteroidetes* (Figure 1B). The possibility of HGT of *zntA* like sequence for other two *Bacteroidetes*, *Chryseobacterium culicis* PMSZPI and *Sphingobacterium siyangense* KMSZPIII, was not taken into consideration as these showed a closest match with a zinc exporting ATPase gene of the complete genome sequence of *Chryseobacterium gleum* ATCC35910 with maximum identity of 92% and 76% respectively. Unlike the *Sphingobacterium* isolates, the G+C contents observed in these isolates matched well with those of the respective genera.

Our study showed intra-phylum evidence for HGT in three other instances. The isolate KMSZP5 was identified as *Bacillus thuringiensis* based on its 16S rRNA gene sequence (Figure 1A) whereas when analysed for its *zntA/cadA/pbrA*-like gene sequences, it exhibited good similarity (94%) with the cadmium-transporting ATPase gene sequence of *Lysinibacillus sphaericus* C3-41 (Table 1) and clustered with *Lysinibacillus* sp. (Figure 1B). The proposition of HGT in the present analysis was established with incongruence between 16S rRNA and ATPase phylogenies. Additionally, the G+C content of the KMSZP5 P_IB_-type ATPase gene was higher (39.8 mol%) than the genomic G+C content of *Bacillus thuringiensis i.e*. 34–35.5 mol% (available at www.ncbi.nlm.nih.gov/genomes/MICROBES). The isolate LONG2 clustered with *Bacillus halmaplus* based on 16S rRNA gene phylogeny (Figure 1A) but the same isolate branched with *Geobacillus kaustophilus* HTA426 using *zntA/cadA/pbrA*-like sequences with 100% bootstrapping (Figure 1B). The phylogenetic incongruence (Figure 1) and the higher G+C content at 45 mol% (considerably higher than the expected 38.6 mol% G+C content of *Bacillus halmapalus*), provided strong evidence for the acquisition of the P_IB_-type ATPase gene through HGT. We confirmed our observations by comparing the mol% G+C content for both species of *Bacillus* as the range for genomic mol% G+C content for the genus *Bacillus* is known to be very wide (32–66%) [35].

The isolate OT6, identified by us as *Pseudomonas koreensis* by 16S rRNA phylogeny, possessed *zntA/cadA/pbrA*-like gene sequences that showed good similarity (92%) with the copper-transporting P_IB_-type ATPase gene of the complete genome of *Providencia rettgeri* (NCBI reference sequence, ZP_06125459.2) with a 100% bootstrap support (Figure 1B). This observation was unaltered even on inclusion of another copper transporting ATPase gene of *Pseudomonas*. The observed mol% G+C content for the isolate OT6 was found to be much lower (48.1%) than is expected for *Pseudomonas* sp. (58–70 mol%) [36]. Thus, in the present investigation, the occurrence of HGT could be demonstrated in six instances (3 inter-phylum and 3 intra-phyla) which were supported by both phylogenetic incongruence and unusual mol% G+C contents.

## Discussion

Bacterial isolates from Domiasiat site displayed remarkable tolerance towards U and other co-occurring heavy metals. The MICs of the isolates towards the tested metals were found to be 2–4 fold higher than those of the respective type strains and as reported for *E.coli* [37]. Among the tested metals, Cd and Pb were found to be the most toxic metals in our study, which is in agreement with earlier findings [37]–[40]. The ability of our subsurface natural isolates of Domiasiat to tolerate and survive against toxic concentrations of U and other heavy metals made us to explore the presence of P_IB_-type ATPases. Further, we analysed the possible dissemination of P_IB_-type ATPase genes through HGT among the natural isolates as HGT was found to be a common occurrence in previously explored metal contaminated sites [17], [18].

In the present study, 32 natural isolates showed a positive signal for a P_IB_-type ATPases gene sequence using gene-specific PCR primers [16]. We obtained *zntA/cadA/pbrA*-like loci from *Bacteroidetes* (*Sphingobacterium* sp. and *Chryseobacterium* sp.) and *Firmicutes* (*Lysinibacillus* sp. and *Paenibacillus* sp.) which deviated from the earlier reported genera [17], [18]. In the case of *Lysinibacillus* sp. and *Paenibacillus* sp., this might be explained by the fact that the amplification primers were designed to target P_IB_-type ATPase genes of *Bacillus* sp. (another genus of *Firmicutes*). Further, it was observed that PCR amplification of P_IB_-type ATPase genes in *Arthrobacter* sp. (RSBA2) and *Pseudomonas* sp. (OT6) showed *copA*-like loci instead of the expected z*ntA/cadA/pbrA*-like loci as reported earlier [18]. Possibly, these deviations might be due to the limited number of control strains used earlier for designing the initial primer sets for P_IB_-type *zntA*/*cadA*/*pbrA*-specific ATPases [16]. In our study, phylogenetic incongruence was observed in six isolates which was also supported by the aberrant G+C contents of their amplified genes. In three instances, acquisition of *zntA*/*cadA*/*pbrA*-like loci by *Bacteroidetes* (KMSDrP1, KMSDrP1, and KMSDrP1) from *Firmicutes* (PSS2) was observed indicating an inter-phylum transfer. The other three isolates KMSZP5, LONG2 and OT6 showed evidences of intra-phylum gene transfer. It is proposed that phylogenetically close microbes were more likely to exchange their genetic material [14] with less likelihood of unveiling the molecular footprint of this transfer in their genomes [41]. Similar results were obtained in our studies where closely related phylogenetic groups were isolated using conditioned enrichment based cultivation method targeting uranium tolerant populations. We might have underestimated the magnitude of HGT of the *zntA/cadA/pbrA*-like loci among the natural isolates as the diversity at the genus level in our study was low. Similar observations were shown by Coombs and Barkay [17] who suggested the use of variety of selection and enrichments methods to obtain diverse isolates for achieving better clarity of HGT in a given environment. With the advent of metagenome and metaproteome applications in microbial ecology and the creation of databases relating 16S rRNA genes and functional genes in uncultured microbes [42]–[45], investigating HGT events among uncultured members of the microbial community should be more feasible in the near future.

Although it is not clear whether HGT events reported in this study occurred before or after U ore mineralization in the selected area, our results clearly indicate the evolution of an ecologically important phenotype suggesting the dissemination of horizontally acquired P_IB_-type ATPase genes among *Firmicutes*, *Bacteroidetes*, and *Proteobacteria* phyla. This study provides an important insight into HGT of P_IB_-type ATPase genes among natural isolates of the U rich deposit located in Domiasiat in North East India.

## Supporting Information

Figure S1 **Phylogenetic analysis of CopA-like cluster of isolates OT6 and RSBA2.** Neighbor-joining cluster analyses were performed for the PCR amplified gene sequences of CopA-like loci from the isolates OT6 and RSBA2 with the gene sequences from complete genomes identified by their gene sequence similarity from NCBI GenBank using BLASTX. Phylogenetic clustering showed that CopA-like loci from OT6 and RSBA2 belonged to the CopA1-gene cluster. The scale bars indicate 0.1 change per amino acid position for P_IB_-type ATPase phylogeny.
(PDF)

Figure S2 **Molecular evidence for horizontal gene transfer among Domiasiat isolates (on exclusion of PMSZPI and KMSZPIII).** (a) 16S rRNA gene and (b) *zntA/cadA/pbrA*-like genes from uranium and heavy metal tolerant isolates obtained from subsurface soils of U rich deposits of Domiasiat were subjected to neighbor-joining analysis. Respective accession numbers are indicated in brackets. P_IB_-type ATPase positive isolates showing HGT are connected by dotted lines. The scale bars indicate 0.05 change per nucleotide position for 16S rRNA gene and 0.1 change per amino acid position for P_IB_-type ATPase phylogeny.
(TIF)

Table S1 **Evidence of HGT for the evolution of** ***zntA/cadA/pbrA*** **-like genes in the U and multi-metal resistant subsurface Domiasiat isolates.**
(DOC)
