# Supplementary figures and images for: Occurrence of Horizontal Gene Transfer of PIB-type ATPase Genes among Bacteria Isolated from the Uranium Rich Deposit of Domiasiat in North East India
Source: PLoS One. 2012 Oct 25;7(10):e48199. doi: 10.1371/journal.pone.0048199 (PMC3485009; doi:10.1371/journal.pone.0048199)

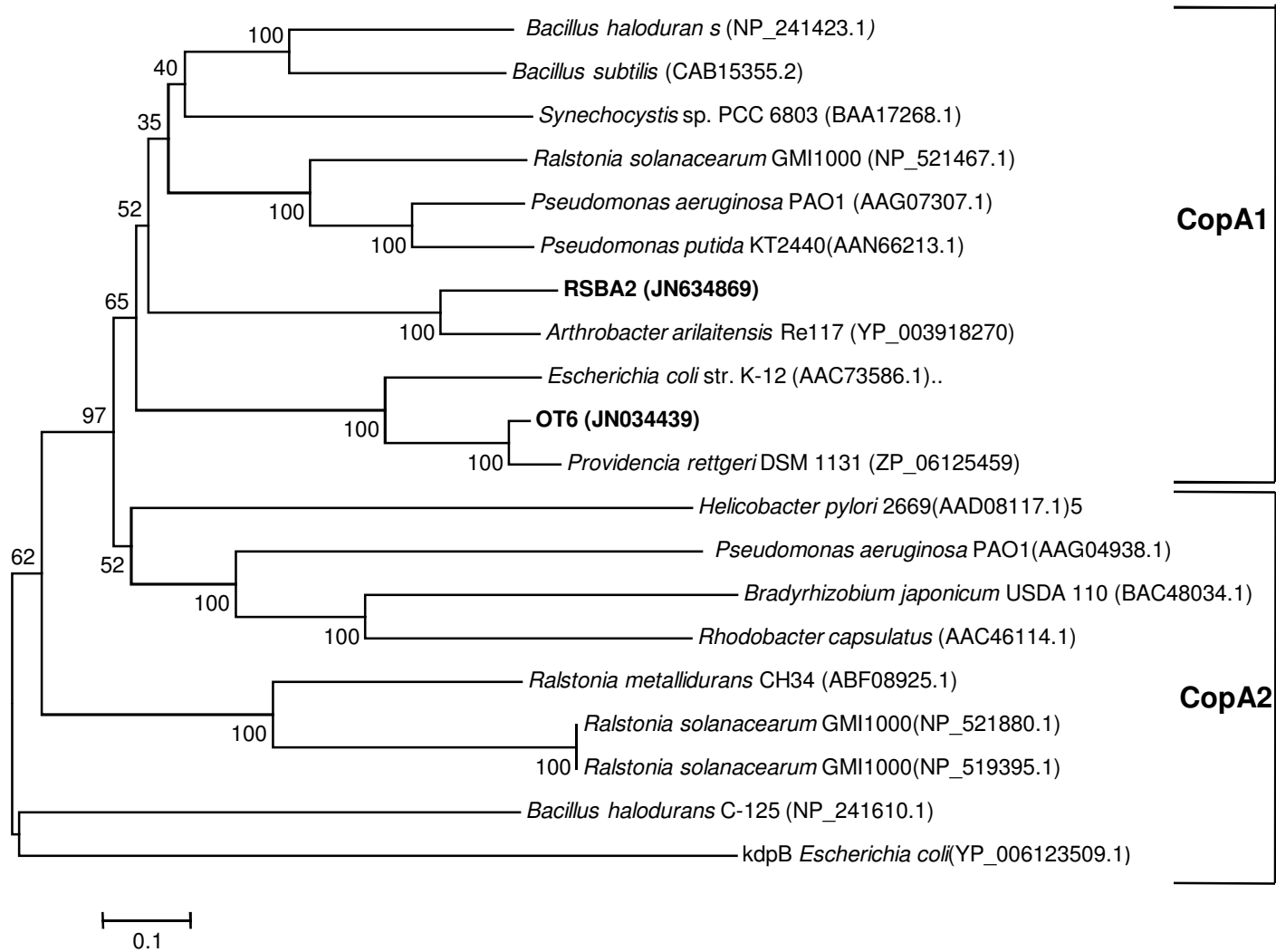

Supplement: Figure S1 — Phylogenetic analysis of CopA-like cluster of isolates OT6 and RSBA2. Neighbor-joining cluster analyses were performed for the PCR amplified gene sequences of CopA-like loci from the isolates OT6 and RSBA2 with the gene sequences from complete genomes identified by their gene sequence similarity from NCBI GenBank using BLASTX. Phylogenetic clustering showed that CopA-like loci from OT6 and RSBA2 belonged to the CopA1-gene cluster. The scale bars indicate 0.1 change per amino acid position for PIB-type ATPase phylogeny. (PDF) [file pone.0048199.s001.pdf]

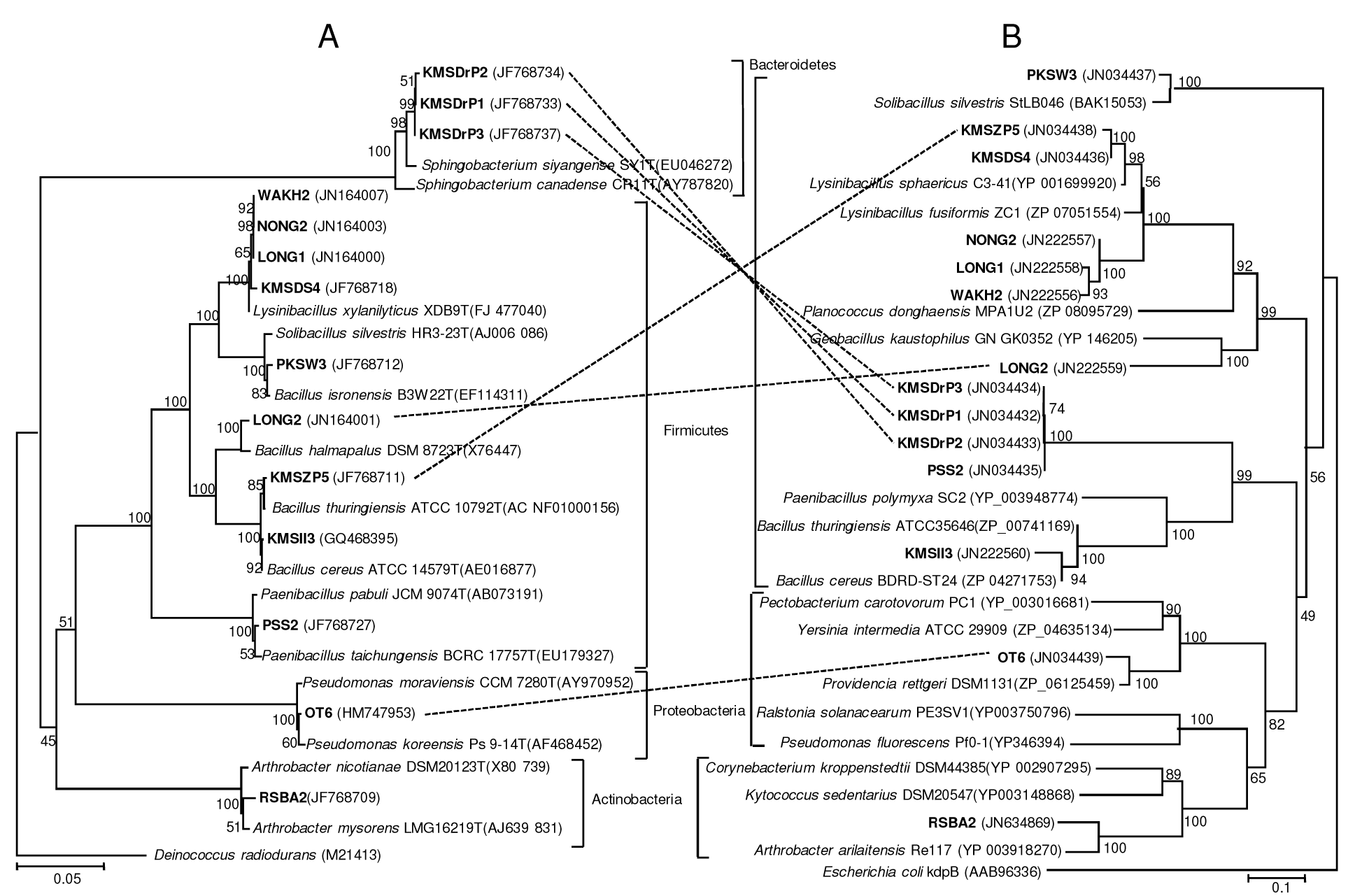

Supplement: Figure S2 — Molecular evidence for horizontal gene transfer among Domiasiat isolates (on exclusion of PMSZPI and KMSZPIII). (a) 16S rRNA gene and (b) zntA/cadA/pbrA-like genes from uranium and heavy metal tolerant isolates obtained from subsurface soils of U rich deposits of Domiasiat were subjected to neighbor-joining analysis. Respective accession numbers are indicated in brackets. PIB-type ATPase positive isolates showing HGT are connected by dotted lines. The scale bars indicate 0.05 change per nucleotide position for 16S rRNA gene and 0.1 change per amino acid position for PIB-type ATPase phylogeny. (TIF) [file pone.0048199.s002.tif]
