# Supplementary material for: Occurrence of Horizontal Gene Transfer of PIB-type ATPase Genes among Bacteria Isolated from the Uranium Rich Deposit of Domiasiat in North East India
Source: PLoS One. 2012 Oct 25;7(10):e48199. doi: 10.1371/journal.pone.0048199 (PMC3485009; doi:10.1371/journal.pone.0048199)
Supplement: Table S1 — Evidence of HGT for the evolution of zntA/cadA/pbrA -like genes in the U and multi-metal resistant subsurface Domiasiat isolates. (DOC) [file pone.0048199.s003.doc]

**Table S1**: **Evidence of HGT for the evolution of z*ntA/cadA/pbrA*-like genes among**

**the uranium and multi-metal resistant subsurface Domiasiat bacterial isolates**

| Isolates | Evidence of HGT supported by | |
| --- | --- | --- |
|  | 1. Phylogenetic incongruence | 1. Unusual G+C content |
| *Sphingobacterium siyangense* KMSDrP1 | Yes | Yes |
| *Sphingobacterium siyangense* KMSDrP2 | Yes | Yes |
| *Sphingobacterium siyangense* KMSDrP3 | Yes | Yes |
| *Bacillus thuringiensis* KMSZP5 | Yes | Yes ^a^ |
| *Bacillus halmapalus* LONG2 | Yes | Yes ^a^ |
| *Pseudomonas koreensis* OT6 | Yes | Yes |

^a^ Abberant G+C was observed when compared with the same species as otherwise the

genus *Bacillus* has large range of DNA G+C content.
